# Persistent Postmastectomy Pain: A Comparison of Diagnosis and Patient-reported Outcome Measures in 6988 Patients

**DOI:** 10.1097/GOX.0000000000007517

**Published:** 2026-03-06

**Authors:** Danielle H. Rochlin, Jacob Levy, Minji Kim, Lillian Boe, Babak J. Mehrara, Jonas A. Nelson

**Affiliations:** From the * Plastic and Reconstructive Surgery Service, Department of Surgery, Memorial Sloan Kettering Cancer Center, New York, NY; † Department of Epidemiology and Biostatistics, Memorial Sloan Kettering Cancer Center, New York, NY.

## Abstract

**Background:**

The purpose of this study was to assess the utility of the BREAST-Q in identifying patients with persistent postmastectomy pain (PPMP) and to determine predictors of pain among a large reconstructive cohort.

**Methods:**

We retrospectively reviewed BREAST-Q physical well-being of the chest (PWBC) scores for patients who underwent breast reconstruction from 2010–2023. PPMP was defined by an International Classification of Diseases diagnosis of pain 3 months to 2 years after mastectomy. Linear regression modeled the association between PWBC score and PPMP, and separately modeled associations with demographic and clinical covariates. Multivariable linear mixed-effects regression was used to analyze PWBC scores over time.

**Results:**

A total of 6988 patients (implant N = 5466; autologous N = 1522) had at least 1 PWBC score preoperatively or 1–5 years postoperatively. PPMP (3.2% of patients) was associated with a lower PWBC score (β = −14, *P* < 0.001). Factors associated with greater odds of PPMP were marital status, number of psychiatric diagnoses, chemotherapy, and radiation. At 1–2 years postoperatively, factors associated with a lower PWBC score included Asian race, Hispanic ethnicity, radiation, and axillary lymph node dissection. Autologous reconstruction demonstrated more favorable long-term PWBC scores compared with implant-based reconstruction.

**Conclusions:**

PPMP was associated with worse PWBC scores. Radiation was the only common predictor of both PPMP and PWBC scores. This correlation, along with differences in predictors, suggests that the BREAST-Q captures some, but not all, elements of postmastectomy pain. Additional validated measures are needed to measure chronic postoperative pain in the breast cancer population.

**Takeaways**

**Question:** Can the BREAST-Q identify patients with persistent postmastectomy pain (PPMP)?

**Findings:** PPMP was associated with worse BREAST-Q physical well-being of the chest scores. Radiation was the only common predictor of both PPMP and physical well-being of the chest scores.

**Meaning:** The BREAST-Q captures some but not all elements of postmastectomy pain. Additional validated measures are needed to measure chronic postoperative pain in the breast cancer population.

## INTRODUCTION

Breast pain among breast cancer survivors is common and morbid. Persistent postmastectomy pain (PPMP) is defined as chronic pain in the anterior chest and/or upper extremity that persists for at least 3 months after mastectomy.^1–3^ Estimates of PPMP prevalence range from 10% to 50%, depending on the definition of PPMP that is applied.^4–9^ Women with PPMP experience higher rates of depression and anxiety, difficulties with sleep, physical impairment, reduced ability to complete activities of daily living and work, and negatively impacted personal relationships; these outcomes contribute to worse quality of life.^7,8,10–14^ PPMP has also been linked to higher rates of medication consumption and is a risk factor for opioid addiction among breast cancer survivors.^15,16^ These sequelae are most acute among racial and ethnic minorities, who have a greater risk of developing PPMP.^17,18^

Despite the extensive literature that has been published to date on the topic, PPMP remains a poorly understood problem due to 2 key shortcomings of prior studies. First, the leading studies on PPMP either do not include women who underwent breast reconstruction or do not account for differences in reconstructive methods in their analyses.^7,19^ This is problematic given that 40%–60% of women nationwide who undergo mastectomy elect to pursue breast reconstruction,^20–22^ and also because of the potential connection between reconstructive technique and postoperative pain.^23,24^ Second, studies to date use inconsistent and nonvalidated measures to quantify PPMP prevalence and severity. These questionnaires have taken the form of de novo patient-reported outcome measures (PROMs), Likert scales, numerical rating scales, short-form health surveys, and visual analog scales, none of which are validated for chronic breast pain and therefore raise questions regarding validity, reliability, and responsiveness.^5,7,8,13,14,19^

The purpose of this study was to assess the utility of the BREAST-Q in identifying patients with PPMP. The BREAST-Q physical well-being of the chest (PWBC) module is a widely used PROM for chest morbidity that was designed and validated among breast reconstruction patients,^25,26^ demonstrating psychometric soundness across multiple studies in terms of construct validity, internal reliability, and sensitivity to change.^23,24,27^ However, the BREAST-Q has not been tested as a measure of chronic pain. Our primary objective was to assess the correlation between BREAST-Q PWBC scores and PPMP in women who underwent breast reconstruction. Our secondary objective was to define the prevalence and risk factors for PPMP in a large longitudinal reconstructive cohort.

## METHODS

### Study Design

With institutional review board approval, we performed a retrospective cohort study of patients who underwent implant-based or autologous reconstruction at Memorial Sloan Kettering Cancer Center between January 2010 and May 2023. All implant-based reconstructions were staged tissue expander-to-implant reconstructions, whereas autologous reconstructions included immediate and delayed reconstructions. Autologous reconstructions involved free tissue transfer with deep inferior epigastric perforator, muscle-sparing transverse rectus abdominis myocutaneous, superficial inferior epigastric artery, profunda artery perforator, diagonal upper gracilis, superior gluteal artery perforator, or latissimus flaps. Female patients aged 18 years or older were included in this study if they had at least 1 BREAST-Q PWBC score preoperatively and/or up to 5 years postoperatively. The BREAST-Q is routinely administered to patients at our institution in conjunction with preoperative and postoperative outpatient clinic visits via an electronic platform. PWBC score is based on patients’ responses to 10 questions about the frequency of discomfort in the breast area, difficulty sleeping due to discomfort, difficulty lifting or moving their arms, and pain in the muscles of the chest.^25^ “Discomfort” refers to pulling, nagging, sharp, aching, and/or throbbing pains, in addition to tightness or tenderness. The raw sum is converted to a 0–100 scaled score, with higher scores indicating better outcomes. The PWBC module also includes an item about lymphedema symptoms that was excluded from our study.

### Variable Selection

We queried patients’ electronic medical records to identify those with a diagnosis of pain based on the following International Classification of Diseases 9th Edition (ICD-9) and 10th Edition (ICD-10) codes: chronic postoperative pain (ICD-9 338.28, ICD-10 G89.28), nerve pain (ICD-9 729.2, ICD-10 M79.2), myofascial pain (ICD-9 729.1, ICD-10 M79.10, M79.18), and mastodynia (ICD-9 611.71, ICD-10 N64.4). We selected patients with a date of diagnosis (ie, date of ICD code entry) between 3 months and 2 years postoperatively. For those who underwent staged surgery, the index surgery was defined as the date of tissue expander placement. PPMP was defined by an ICD diagnosis of pain that was first noted during this timeframe. Patients with a preoperative ICD diagnosis of pain were excluded.

Other demographic and clinical variables of interest included age at surgery, body mass index (BMI), race (White, Asian, Black, other/unknown), ethnicity (not Hispanic or Hispanic), marital status (married/partnered, separated or divorced, or single), tobacco use (never, current, or former), number of psychiatric diagnoses, neoadjuvant or adjuvant chemotherapy, neoadjuvant or adjuvant radiation therapy, type of reconstruction (implant or autologous), laterality (unilateral or bilateral), timing of reconstruction, sentinel lymph node biopsy, and axillary lymph node dissection (ALND). For patients with tissue expanders, additional variables included the use of acellular dermal matrix (ADM), the plane of pocket dissection (subpectoral or prepectoral), and receipt of a preoperative peripheral nerve block.

### Statistical Analysis

Patient demographics, clinical characteristics, and BREAST-Q scores were summarized descriptively, with percentages for categorical variables and medians with interquartile ranges (IQRs) for continuous variables. Categorical and continuous variables were compared with the Wilcoxon rank-sum test and Pearson χ^2^ test, respectively. BREAST-Q scores at all time points were compared with the Wilcoxon rank-sum test. False discovery rate correction was applied to account for multiple testing. Linear regression modeled the relationship between PWBC scores at 1–2 years postoperatively and postoperative ICD diagnoses of breast pain for both the overall cohort and the implant subgroup. PWBC score at 1–2 years postoperatively was defined as a patient’s latest available score within the first or second year after surgery. Additional linear regression evaluated the association between patient demographic and clinical variables and PWBC score at 1–2 years postoperatively, and with postoperative pain diagnosis. Multivariable linear mixed-effects regression for implant and autologous subgroups modeled the relationship between PWBC scores over time, where time and demographic and clinical variables were included as fixed effects, and a random intercept was estimated for each patient. A 3-point change in PWBC score, rounded to the nearest integer, was considered clinically significant based on the established minimally clinically important difference.^28^ *P* values of less than 0.05 were considered statistically significant. Statistical analyses were performed using R version 4.3.2.

## RESULTS

A total of 6988 patients (implant N = 5466; autologous N = 1522) had at least 1 PWBC score preoperatively (N = 3285) or at 1 (N = 4009), 2 (N = 2927), 3 (N = 2887), 4 (N = 1942), or 5 (N = 1574) years postoperatively (Table 1). Overall, median (IQR) age at surgery was 49 (43, 56) years, and median (IQR) BMI was 23.7 (21.1, 27.3) kg/m^2^. Most patients were White (76%; Asian 7.7%, Black 8.4%), not Hispanic or Latino (89%; Hispanic or Latino 7.4%), married or partnered (73%), and never (73%) or former (24%) tobacco users. Rates of neoadjuvant and/or adjuvant chemotherapy and radiation therapy were 52% and 22%, respectively. Rates of sentinel lymph node biopsy and ALND were 85% and 19%, respectively. Among patients who underwent implant-based reconstruction, 26% involved ADM, 29% received a peripheral nerve block, and 87% had a subpectoral pocket. Table 2 displays a complete summary of patient-level variables.

**Table 1.** BREAST-Q Scores—PWBC

Characteristic	N	Overall, N = 6988^*^	N	Autologous, N = 1522^*^	N	Implant, N = 5466^*^	*P* ^†^	*q* ^‡^
Preoperative	3285	85 (72, 100)	805	77 (64, 91)	2480	85 (74, 100)	<0.001	<0.001
One-year postoperative	4009	76 (64, 91)	882	76 (63, 91)	3127	76 (64, 91)	0.5	0.6
Two-year postoperative	2927	77 (64, 91)	579	77 (64, 92)	2348	77 (64, 91)	0.5	0.6
One- to 2-year postoperative	4959	76 (64, 91)	1038	76 (64, 92)	3921	76 (64, 91)	0.3	0.6
Three-year postoperative	2887	76 (64, 85)	743	68 (59, 80)	2144	77 (64, 91)	<0.001	<0.001
Four-year postoperative	1942	77 (64, 92)	226	80 (64, 92)	1716	77 (64, 92)	0.6	0.6
Five-year postoperative	1574	80 (66, 92)	126	81 (68, 100)	1448	77 (64, 92)	0.073	0.2

* Median (IQR).

† Wilcoxon rank sum test.

‡ False discovery rate correction for multiple testing.

**Table 2.** Patient-level Variables

Characteristic	Overall, N = 6988^*^	Autologous, N = 1522^*^	Implant, N = 5466^*^	*P* ^†^	*q* ^‡^
Age at surgery, y	49 (43, 56)	51 (44, 57)	49 (42, 56)	<0.001	<0.001
BMI, kg/m^2^	23.7 (21.1, 27.3)	26.6 (23.5, 29.9)	22.9 (20.7, 26.2)	<0.001	<0.001
Race				<0.001	<0.001
Asian	541 (7.7)	124 (8.1)	417 (7.6)		
Black	589 (8.4)	196 (13)	393 (7.2)		
Other/unknown	520 (7.4)	155 (10)	365 (6.7)		
White	5338 (76)	1047 (69)	4291 (79)		
Ethnicity				<0.001	<0.001
Hispanic or Latino	517 (7.4)	148 (9.7)	369 (6.8)		
Not Hispanic	6236 (89)	1309 (86)	4927 (90)		
Unknown	235 (3.4)	65 (4.3)	170 (3.1)		
Marital status				0.6	0.6
Married/partner	5081 (73)	1092 (72)	3989 (73)		
Separated/divorced/widowed	666 (9.5)	154 (10)	512 (9.4)		
Single	1241 (18)	276 (18)	965 (18)		
Tobacco use				0.6	0.6
Current	163 (2.3)	30 (2.0)	133 (2.4)		
Former	1698 (24)	368 (24)	1330 (24)		
Never	5127 (73)	1124 (74)	4003 (73)		
No. psychiatric diagnoses	2 (0, 2)	2 (0, 3)	2 (0, 2)	0.3	0.3
Chemotherapy	3633 (52)	1148 (75)	2485 (45)	<0.001	<0.001
Radiation therapy	1541 (22)	442 (29)	1099 (20)	<0.001	<0.001
Laterality				<0.001	<0.001
Bilateral	4172 (60)	715 (47)	3457 (63)		
Unilateral	2816 (40)	807 (53)	2009 (37)		
Timing of reconstruction				<0.001	<0.001
Delayed	790 (11)	670 (44)	120 (2.2)		
Immediate	6198 (89)	852 (56)	5346 (98)		
ALND	1362 (19)	396 (26)	966 (18)	<0.001	<0.001
SLNB	5952 (85)	1258 (83)	4694 (86)	0.002	0.002
ADM				<0.001	<0.001
No	4058 (58)	0 (0)	4058 (74)		
Unknown/not applicable	1522 (22)	1522 (100)	0 (0)		
Yes	1408 (20)	0 (0)	1408 (26)		
Block				<0.001	<0.001
No	3899 (56)	0 (0)	3899 (71)		
Not applicable	1522 (22)	1522 (100)	0 (0)		
Yes	1567 (22)	0 (0)	1567 (29)		
Pocket dissection				<0.001	<0.001
Not applicable	1522 (22)	1522 (100)	0 (0)		
Prepectoral	693 (9.9)	0 (0)	693 (13)		
Subpectoral	4773 (68)	0 (0)	4773 (87)		

* Median (IQR); n (%).

† Wilcoxon rank sum test; Pearson χ^2^ test.

‡ False discovery rate correction for multiple testing.

SLNB, sentinel lymph node biopsy.

Among the 4959 patients with a PWBC score at 1–2 years postoperatively, 159 (3.2%) had an ICD diagnosis of pain within 3 months to 2 years postoperatively. The median (IQR) time between surgery and ICD diagnosis was 0.86 (0.48, 1.41) years. (**See table, Supplemental Digital Content 1**, which displays the association between ICD pain diagnosis and BREAST-Q scores, https://links.lww.com/PRSGO/E681.) An ICD diagnosis of pain was associated with a lower PWBC score at 1–2 years postoperatively (β = −14, 95% confidence interval [CI] −17 to −11, *P* < 0.001). (**See table, Supplemental Digital Content 2**, which displays the univariable linear regression model for PWBC BREAST-Q at 1–2 y, https://links.lww.com/PRSGO/E682.)

Factors that were associated with an ICD diagnosis of pain between 3 months and 2 years postoperatively were marital status of separated, divorced, or widowed (odds ratio [OR] 1.64, 95% CI 1.09–2.41, *P* = 0.013); number of psychiatric diagnoses (OR 1.31, 95% CI 1.19–1.43, *P* < 0.001); chemotherapy (OR 1.69, 95% CI 1.19–2.42, *P* = 0.004); and radiation (OR 1.54, 95% CI 1.11–2.13, *P* = 0.010) (Table 3). Patients with implant-based reconstruction had a lower odds of a pain diagnosis compared to patients with autologous reconstruction (OR 0.55, 95% CI 0.38–0.79, *P* = 0.001). In a subgroup analysis of patients who underwent implant-based reconstruction, additional covariates of ADM, block, and plane of pocket dissection were not significantly associated with the odds of a pain diagnosis (Table 4).

**Table 3.** Multivariable Logistic Regression Model for ICD Diagnosis of Pain 3 Months to 2 Years After Surgery Among All Patients (Implant and Autologous) (N = 6988)

Characteristic	OR	95% CI	*P*
(Intercept)	0.01	0.00–0.03	<0.001
Age at surgery, y	1.00	0.99–1.02	0.8
BMI, kg/m^2^	1.00	0.97–1.03	0.8
Race			
White	—	—	
Asian	0.99	0.52–1.73	>0.9
Black	1.43	0.90–2.19	0.12
Other/unknown	1.23	0.74–1.97	0.4
Ethnicity			
Not Hispanic	—	—	
Hispanic or Latino	1.53	0.95–2.38	0.070
Unknown	0.83	0.32–1.77	0.7
Marital status			
Married/partner	—	—	
Separated/divorced/widowed	1.64	1.09–2.41	0.013
Single	1.03	0.71–1.48	0.9
Tobacco use			
Never	—	—	
Current	0.90	0.37–1.88	0.8
Former	1.03	0.74–1.40	0.9
No. psychiatric diagnoses	1.31	1.19–1.43	<0.001
Chemotherapy	1.69	1.19–2.42	0.004
Radiation therapy	1.54	1.11–2.13	0.010
Laterality			
Bilateral	—	—	
Unilateral	1.02	0.76–1.37	0.9
Timing of reconstruction			
Delayed	—	—	
Immediate	1.56	1.00–2.50	0.056
ALND	1.16	0.83–1.60	0.4
SLNB	0.88	0.61–1.30	0.5
Reconstruction method			
Autologous	—	—	
Implant	0.55	0.38–0.79	0.001

SLNB, sentinel lymph node biopsy.

**Table 4.** Multivariable Logistic Regression Model for ICD Diagnosis of Pain 3 Months to 2 Years After Surgery Among Implant Patients (N = 5466)

Characteristic	OR	95% CI	*P*
(Intercept)	0.01	0.00–0.04	<0.001
Age at surgery, y	1.00	0.98–1.02	>0.9
BMI, kg/m^2^	1.00	0.96–1.04	>0.9
Marital status			
Married/partner	—	—	
Separated/divorced/widowed	1.81	1.11–2.87	0.014
Single	0.98	0.61–1.51	>0.9
No. psychiatric diagnoses	1.31	1.18–1.46	<0.001
Chemotherapy	1.62	1.08–2.44	0.019
Radiation therapy	1.77	1.20–2.62	0.004
ADM			
Yes	—	—	
No	0.83	0.55–1.29	0.4
Block			
No	—	—	
Yes	0.80	0.53–1.18	0.3
Pocket dissection			
Subpectoral	—	—	
Prepectoral	0.48	0.21–0.97	0.055

Factors that were associated with worse PWBC score at 1–2 years postoperatively based on clinical and statistical significance included Asian race (β = −4.0, 95% CI −5.9 to −2.0, *P* < 0.001), Hispanic or Latino ethnicity (β = −4.9, 95% CI −6.9 to −2.8, *P* < 0.001), radiation (β = −5.0, 95% CI −6.4 to −3.6, *P* < 0.001), and ALND (β = −4.2, 95% CI −5.6 to −2.8, *P* < 0.001). In a subgroup analysis of patients who underwent implant-based reconstruction, covariates with clinical and statistical significance were similar to those of the total cohort regression. (**See table, Supplemental Digital Content 3**, which displays the multivariable linear regression model for PWBC BREAST-Q at 1–2 y among implant patients [N = 3921], https://links.lww.com/PRSGO/E683.)

Prepectoral pocket dissection was associated with a higher PWBC score (β = 4.8, 95% CI 2.9–6.7, *P* < 0.001). ADM and peripheral nerve block demonstrated no significant association.

**Fig. 1.**
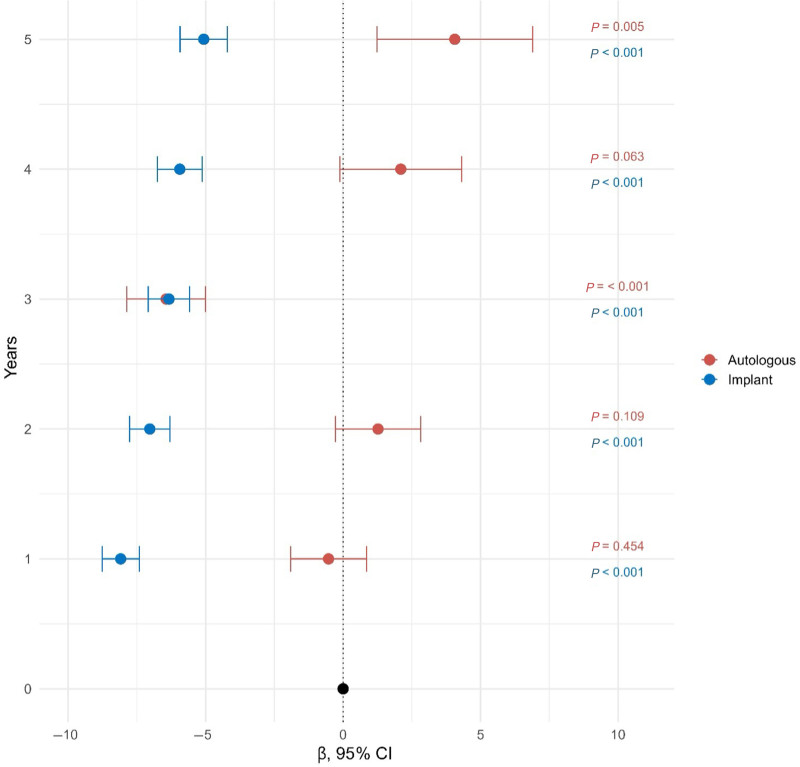
Adjusted PWBC score over time compared with preoperative score.

Multivariable linear mixed-effects regression included all covariates from the prior linear regression analyses, as well as time as the fixed effect, and estimated a random intercept for each patient. Compared with a preoperative baseline, PWBC scores for patients who underwent implant-based reconstruction were significantly worse at all postoperative time points (*P* < 0.001), with coefficients ranging from −8.1 to −5.1. For patients who underwent autologous reconstruction, PWBC scores were significantly lower than the preoperative baseline at 3 years postoperatively (β = −6.4, 95% CI −7.9 to −5.0, *P* < 0.001) and significantly higher than the preoperative baseline at 5 years postoperatively (β = 4.1, 95% CI 1.2–6.9, *P* = 0.005) (Fig. 1). (**See table, Supplemental Digital Content 4**, which displays the multivariable linear mixed-effects model for PWBC over time among implant patients [N = 5466], https://links.lww.com/PRSGO/E684.) (**See table, Supplemental Digital Content 5**, which displays the multivariable linear mixed-effects model for PWBC over time among autologous patients [N = 1522], https://links.lww.com/PRSGO/E685.)

## DISCUSSION

In this analysis of 6988 women who underwent implant-based or autologous breast reconstruction, we demonstrate that PPMP—as defined by an ICD diagnosis of pain 3 months to 2 years postoperatively—and BREAST-Q PWBC scores were associated yet had different predictors. PPMP was significantly associated with worse PWBC scores at 1–2 years postoperatively with a magnitude that was more than 4 times the minimal clinically important difference. PPMP was associated with marital status (separated, divorced, or widowed), the number of psychiatric diagnoses, chemotherapy, and radiation. PWBC scores at 1–2 years postoperatively were worse among Asian and Hispanic/Latino patients, as well as among patients who had undergone radiation therapy and/or ALND; PWBC scores were more favorable for prepectoral implant placement. Implant-based reconstruction was associated with a lower odds of PPMP but consistently lower PWBC scores during the 5-year follow-up period.

The trajectory of PWBC scores among autologous patients likely reflects the long recovery course of free flap reconstruction. At 3 years, lower scores compared to baseline may reflect persistent donor-site morbidity, surgical revisions, and the effects of adjuvant therapies such as radiation. By 5 years, however, many patients experience sustained resolution of subacute postoperative symptoms, adaptation to their reconstruction, and improved chest wall comfort compared with the preoperative baseline, when many were recovering from cancer treatment and/or the sequelae of mastectomy. Prior long-term studies of autologous reconstruction have similarly demonstrated durable improvements in satisfaction and quality of life, often approaching and reaching preoperative levels.^24,27^

Though pain diagnosis and PWBC score were correlated, all significant predictors of these outcomes differed with the exception of the common predictor of radiation. Prior studies have enumerated a more encompassing and overlapping list of predictors. Lim et al^29^ synthesized the existing literature on persistent pain after breast surgery in a recent meta-analysis, concluding that major risk factors include higher BMI, anxiety, depression, diabetes, smoking, preoperative and acute postoperative pain, reoperation, radiation, and ALND. Of note, of the 126 breast surgery studies in this meta-analysis, fewer than 25% mentioned inclusion of reconstruction patients in the study cohort, and only 3 considered the impact of reconstruction in the analysis. Similar to our findings, published predictors of lower BREAST-Q scores include radiation, ALND, the number of psychiatric diagnoses, subpectoral implant placement, and race and ethnicity.^27,30–36^ Prior studies report higher rates of PPMP among women of racial and ethnic minority groups,^37^ potentially due to differences in biological, clinical, and social factors^38–40^; however, race and ethnicity were not significant predictors of pain diagnosis in our analysis. Race and ethnicity as significant predictors of PWBC scores but not PPMP suggest that PWBC scores and ICD diagnoses of pain may be capturing different outcomes despite their strong correlation. These discrepancies may reflect cultural and social factors that shape how symptoms are experienced, communicated, or documented, as well as systemic differences in healthcare access and potential implicit bias in pain assessment. Further research is needed to determine whether these disparities reflect differences in true pain burden or differences in how pain is recognized and addressed. Similarly, implant-based reconstruction demonstrated a lower odds of PPMP yet less favorable PWBC scores over time, indicating that PWBC as measured by the BREAST-Q may be more encompassing than postoperative pain.

The ultimate goal of this line of research is to identify patients who are at risk of developing PPMP and triage them to appropriate interventions. For instance, predictors of chronic pain identified in this study may be used to determine which patients may benefit from early referral to supportive care, such as pain management and/or physical therapy. Greater specificity in the diagnosis of PPMP would improve our ability to both triage patients and more narrowly define the prevalence of PPMP. The International Association for the Study of Pain defines pain based on 3 pathophysiological categories that can be applied to PPMP in the setting of reconstruction: nociceptive, a response to stimuli associated with direct tissue damage (eg, secondary musculoskeletal pain, capsular contracture, costochondritis); neuropathic, resulting from injury to or disease of the somatosensory nervous system (eg, stretch, direct mechanical or compressive nerve injury, or neuritis/neuropathy); and nociplastic, arising from altered nociception without evidence of any noxious stimulus (eg, fibromyalgia, complex regional pain syndrome).^2,3^ The appropriateness of interventions varies based on the pathophysiological category. For instance, patients with neuropathic PPMP, often referred to as postmastectomy pain syndrome, may benefit from a nerve block or resection of an intercostal neuroma, whereas nociceptive PPMP patients may be more responsive to nonsteroidal anti-inflammatory drugs or capsulectomy. This study represented a step toward both predicting and identifying reconstruction patients at risk for PPMP, though further specificity in the diagnosis of PPMP is needed to facilitate treatment.

The key novelty of this study is the consideration of the BREAST-Q PWBC module in the context of PPMP. There is currently no validated measure for the diagnosis and assessment of PPMP. The BREAST-Q was designed to assess patients’ satisfaction and quality of life in relation to their reconstruction.^25^ Our methodology of comparing PWBC scores with an ICD diagnosis of postoperative pain suggests that the BREAST-Q PWBC score correlates with PPMP to some degree, although differences in predictors indicate outstanding differences. Although pain-related items constitute an important component of the PWBC module, the score also reflects other domains such as range of motion and chest wall discomfort, capturing functional and quality-of-life limitations that extend beyond pain diagnosis alone. Although the gold standard would be to develop and validate a novel PROM specific to the breast cancer population, a more feasible step in the short term would be to explore measures that are validated in the chronic pain population, such as the Patient-Reported Outcomes Measurement Information System (PROMIS) from the National Institutes of Health, which may demonstrate improved overlap with ICD pain diagnoses compared with the BREAST-Q. PROMIS consists of item banks for various health-related quality-of-life domains that are validated for use in the general population and in individuals living with chronic conditions; specific item banks measure pain intensity, pain interference, and pain quality, including nociceptive versus neuropathic pain quality.^41^ PROMIS data regarding nociceptive versus neuropathic pain quality would additionally enhance our ability to triage patients to appropriate therapeutic interventions.

This study has both strengths and limitations. In addition to the novelty of examining PPMP through the lens of the BREAST-Q, our study used a large cohort with follow-up across a 5-year timeframe. We excluded patients with an ICD diagnosis of chronic postoperative pain, nerve pain, myofascial pain, or mastodynia if the diagnosis occurred preoperatively, before 3 months postoperatively, or after 2 years postoperatively, with the latter exclusion criteria intended to omit patients with acute postoperative pain or pain due to other causes, respectively. Although unlikely, it is possible that a patient had an unrelated procedure or clinical event within the 3-month to 2-year postoperative timeframe that precipitated a pain diagnosis. More likely, our definition of PPMP prevalence via ICD diagnosis likely underestimates the true prevalence of PPMP in this population, as patients may have been lost to follow-up during the study timeframe. We defined PPMP by ICD diagnosis of pain, though we acknowledge that this is an approximation and that definitions of PPMP vary throughout the literature.^42,43^ In addition, though routinely used as a tool to query electronic medical records and larger claims databases, ICD diagnoses may misrepresent or underrepresent true clinical diagnoses.^44,45^ This study was retrospective with gaps in longitudinal data at the patient level; thus, longitudinal data are presented in aggregate and may miss patient-specific trends. It is possible that pain is related to capsular contracture, though this variable was not consistently coded in our dataset and was not included in our analysis. Finally, although our analysis of prepectoral versus subpectoral implant placement provides valuable insights, the relatively small proportion of prepectoral cases (9.9%) in our cohort limits generalizability.

## CONCLUSIONS

Postmastectomy pain was strongly associated with worse PWBC scores. However, radiation was the only common predictor of both pain diagnosis and PWBC score. These differences in predictors suggest that the BREAST-Q captures some, but not all, elements of PPMP. Research on the measurement of PPMP with validated and more specific PROMs is warranted to improve our ability to diagnose and manage this complex condition.

## DISCLOSURES

The authors have no financial interest to declare in relation to the content of this article. Dr. Mehrara is the recipient of investigator-initiated research grants from Pfizer, Integra, and Regeneron and has received royalty payments from Elsevier; he has also served as a consultant for Mediflix Corp.

## ACKNOWLEDGMENTS

This research was supported in part by the Cancer Center Support Grant P30 CA008748, which supports the research infrastructure at Memorial Sloan Kettering Cancer Center. The content is solely the responsibility of the authors and does not necessarily represent the official views of the National Institutes of Health.

## Supplementary Material


