# Supplementary material for: Persistent Postmastectomy Pain: A Comparison of Diagnosis and Patient-reported Outcome Measures in 6988 Patients
Source: Plast Reconstr Surg Glob Open. 2026 Mar 6;14(3):e7517. doi: 10.1097/GOX.0000000000007517 (PMC12966117; doi:10.1097/GOX.0000000000007517)
Supplement: Supplementary file 1 [file gox-14-e7517-s001.pdf]

Supplemental Digital Content 1. Association between ICD Pain Diagnosis and BREAST-Q Scores

**Characteristic**	**N**	**Overall**, N = 6,988[1]	**N**	**No Pain**, N = 6,766[1]	**N**	**Pain**, N = 222[1]	**p-value**[2]
**Physical Well-Being of the Chest BREAST-Q at 1-2Yrs**	4,959	76 (64, 91)	4,800	77 (64, 91)	159	63 (50, 77)	<0.001
**Time between surgery and postop ICD Diagnosis (Years)**	222	0.86 (0.48, 1.41)			222	0.86 (0.48, 1.41)	

[1]Median (IQR)

[2]Wilcoxon rank sum test
