# Supplementary material for: Persistent Postmastectomy Pain: A Comparison of Diagnosis and Patient-reported Outcome Measures in 6988 Patients
Source: Plast Reconstr Surg Glob Open. 2026 Mar 6;14(3):e7517. doi: 10.1097/GOX.0000000000007517 (PMC12966117; doi:10.1097/GOX.0000000000007517)
Supplement: Supplementary file 2 [file gox-14-e7517-s002.pdf]

Supplemental Digital Content 2. Univariable Linear Regression Model for Physical Well-Being of the Chest BREAST-Q at 1-2Yrs

**Characteristic**	**Beta**	**95% CI[1]**	**p-value**
**(Intercept)**	76	75, 77	<0.001
**Postop Pain (within 2 years)**	-14	-17, -11	<0.001

[1]CI = Confidence Interval
