# Supplementary material for: Persistent Postmastectomy Pain: A Comparison of Diagnosis and Patient-reported Outcome Measures in 6988 Patients
Source: Plast Reconstr Surg Glob Open. 2026 Mar 6;14(3):e7517. doi: 10.1097/GOX.0000000000007517 (PMC12966117; doi:10.1097/GOX.0000000000007517)
Supplement: Supplementary file 3 [file gox-14-e7517-s003.pdf]

Supplemental Digital Content 3. Multivariable Linear Regression Model for Physical Well-Being of the Chest BREAST-Q at 1-2Yrs, Implant Patients (N = 3921)

Characteristic	Beta	95% CI[1]	p-value
**(Intercept)**	91	85, 96	<0.001
**Age at Surgery**	-0.07	-0.13, -0.01	0.022
**BMI**	-0.33	-0.45, -0.21	<0.001
**Race**			
White	—	—	
Asian	-4.5	-6.7, -2.3	<0.001
Black	-1.8	-4.1, 0.46	0.12
Other/Unknown	-1.9	-4.3, 0.50	0.12
**Ethnicity**			
Not Hispanic	—	—	
Hispanic or Latino	-5.0	-7.3, -2.6	<0.001
Unknown	-0.03	-3.1, 3.0	>0.9
**Marital Status**			
Married/Partner	—	—	
Separated/Divorced/Widowed	0.03	-1.9, 2.0	>0.9
Single	0.00	-1.5, 1.5	>0.9
**Smoking**			
Never Smoker	—	—	
Current Smoker	-3.7	-7.4, 0.05	0.053
Former Smoker	-1.4	-2.8, -0.12	0.033
**Number of psychiatric diagnoses**	-1.4	-1.8, -0.95	<0.001
**Chemotherapy**	1.0	-0.27, 2.3	0.12
**Radiation Therapy**	-4.9	-6.6, -3.3	<0.001
**Laterality**			
Bilateral	—	—	
Unilateral	0.23	-0.99, 1.4	0.7
**Timing of Reconstruction**			
Delayed	—	—	
Immediate	-1.1	-5.0, 2.9	0.6
**ALND**	-4.3	-5.9, -2.7	<0.001
**SLNB**	0.87	-0.79, 2.5	0.3
**ADM**			
Yes	—	—	
No	0.12	-1.4, 1.6	0.9
**Block**			
No	—	—	
Yes	-0.39	-1.6, 0.81	0.5
**Pocket Dissection**			
Subpectoral	—	—	
Prepectoral	4.8	2.9, 6.7	<0.001

[1]CI = Confidence Interval
