# Supplementary material for: Persistent Postmastectomy Pain: A Comparison of Diagnosis and Patient-reported Outcome Measures in 6988 Patients
Source: Plast Reconstr Surg Glob Open. 2026 Mar 6;14(3):e7517. doi: 10.1097/GOX.0000000000007517 (PMC12966117; doi:10.1097/GOX.0000000000007517)
Supplement: Supplementary file 4 [file gox-14-e7517-s004.pdf]

Supplemental Digital Content 4. Multivariable Linear Mixed Effects Model for Physical Well-Being of the Chest Over Time, Implant Patients (N = 5466)

Characteristic	Beta	95% CI[1]	p-value
**(Intercept)**	96	92, 100	<0.001
**Time**			
PreOp	—	—	
1Year	-8.1	-8.8, -7.4	<0.001
2Years	-7.0	-7.8, -6.3	<0.001
3Years	-6.3	-7.1, -5.6	<0.001
4Years	-5.9	-6.7, -5.1	<0.001
5Years	-5.1	-5.9, -4.2	<0.001
**Age at Surgery**	-0.05	-0.09, -0.01	0.018
**BMI**	-0.32	-0.41, -0.24	<0.001
**Race**			
White	—	—	
Asian	-4.5	-6.1, -3.0	<0.001
Black	-2.5	-4.1, -0.83	0.003
Other/Unknown	-2.2	-3.9, -0.45	0.013
**Ethnicity**			
Not Hispanic	—	—	
Hispanic or Latino	-3.9	-5.6, -2.2	<0.001
Unknown	0.02	-2.3, 2.4	>0.9
**Marital Status**			
Married/Partner	—	—	
Separated/Divorced/Widowed	-0.56	-2.0, 0.85	0.4
Single	0.16	-0.95, 1.3	0.8
**Smoking**			
Never Smoker	—	—	
Current Smoker	-3.3	-6.0, -0.64	0.015
Former Smoker	-1.0	-2.0, -0.08	0.034
**Number of psychiatric diagnoses**	-1.3	-1.6, -0.97	<0.001
**Chemotherapy**	0.38	-0.56, 1.3	0.4
**Radiation Therapy**	-3.5	-4.7, -2.3	<0.001
**Laterality**			
Bilateral	—	—	
Unilateral	0.33	-0.56, 1.2	0.5
**Timing of Reconstruction**			
Delayed	—	—	
Immediate	0.18	-2.7, 3.0	>0.9
**ALND**	-3.2	-4.4, -2.0	<0.001
**SLNB**	0.29	-0.90, 1.5	0.6
**ADM**			
Yes	—	—	
No	-0.28	-1.4, 0.79	0.6
**Block**			
No	—	—	
Yes	0.32	-0.58, 1.2	0.5
**Pocket Dissection**			

**Characteristic**	**Beta**	**95% CI**[1]	**p-value**
Subpectoral	—	—	
Prepectoral	4.3	2.9, 5.8	<0.001

[1]CI = Confidence Interval
