# Supplementary material for: Persistent Postmastectomy Pain: A Comparison of Diagnosis and Patient-reported Outcome Measures in 6988 Patients
Source: Plast Reconstr Surg Glob Open. 2026 Mar 6;14(3):e7517. doi: 10.1097/GOX.0000000000007517 (PMC12966117; doi:10.1097/GOX.0000000000007517)
Supplement: Supplementary file 5 [file gox-14-e7517-s005.pdf]

Supplemental Digital Content 5. Multivariable Linear Mixed Effects Model for Physical Well-Being of the Chest Over Time, Autologous Patients (N = 1522)

Characteristic	Beta	95% CI[1]	p-value
**(Intercept)**	89	81, 96	<0.001
**Time**			
PreOp	—	—	
1Year	-0.53	-1.9, 0.85	0.5
2Years	1.3	-0.28, 2.8	0.11
3Years	-6.4	-7.9, -5.0	<0.001
4Years	2.1	-0.11, 4.3	0.063
5Years	4.1	1.2, 6.9	0.005
**Age at Surgery**	-0.06	-0.15, 0.03	0.2
**BMI**	-0.21	-0.39, -0.04	0.015
**Race**			
White	—	—	
Asian	-4.1	-7.1, -1.1	0.008
Black	-3.3	-5.8, -0.76	0.011
Other/Unknown	-3.1	-6.0, -0.14	0.040
**Ethnicity**			
Not Hispanic	—	—	
Hispanic or Latino	-3.4	-6.3, -0.44	0.024
Unknown	0.15	-3.8, 4.1	>0.9
**Marital Status**			
Married/Partner	—	—	
Separated/Divorced/Widowed	0.13	-2.5, 2.8	>0.9
Single	-0.17	-2.3, 2.0	0.9
**Smoking**			
Never Smoker	—	—	
Current Smoker	5.2	-0.64, 11	0.081
Former Smoker	0.72	-1.2, 2.6	0.4
**Number of psychiatric diagnoses**	-1.4	-1.9, -0.83	<0.001
**Chemotherapy**	2.8	0.74, 4.8	0.008
**Radiation Therapy**	-4.0	-5.9, -2.0	<0.001
**Laterality**			
Bilateral	—	—	
Unilateral	-0.19	-1.8, 1.5	0.8
**Timing of Reconstruction**			
Delayed	—	—	
Immediate	0.53	-1.2, 2.2	0.5
**ALND**	-2.4	-4.4, -0.43	0.017
**SLNB**	-2.2	-4.4, -0.06	0.044

[1]CI = Confidence Interval
